# The quantity and quality of α‐gal‐specific antibodies differ in individuals with and without delayed red meat allergy

**DOI:** 10.1111/all.12948

**Published:** 2016-07-15

**Authors:** D. Kollmann, B. Nagl, C. Ebner, W. Emminger, S. Wöhrl, C. Kitzmüller, S. Vrtala, A. Mangold, H.‐J. Ankersmit, B. Bohle

**Affiliations:** ^1^ Department of Pathophysiology and Allergy ResearchMedical University of ViennaViennaAustria; ^2^ Allergy Clinic ReumannplatzViennaAustria; ^3^ Allergy Clinic RennwegViennaAustria; ^4^ Allergy Clinic FloridsdorfViennaAustria; ^5^ Department of Internal MedicineMedical University of ViennaViennaAustria; ^6^ Department of Thoracic SurgeryMedical University of ViennaViennaAustria

**Keywords:** allergens and epitopes, food allergy, IgE

## Abstract

**Background:**

IgG to galactose‐α‐1,3‐galactose (α‐gal) are highly abundant natural antibodies (Ab) in humans. α‐Gal‐specific IgE Ab cause a special form of meat allergy characterized by severe systemic reactions 3–7 h after consumption of red meat. We investigated 20 patients who experienced such reactions and characterized their α‐gal‐specific IgE and IgG responses in more detail.

**Methods:**

α‐Gal‐specific IgE was determined by ImmunoCAP. IgE reactivity to meat extract and bovine gamma globulin (BGG) was assessed by immunoblotting and ELISA, respectively. In some experiments, sera were pre‐incubated with α‐gal or protein G to deplete IgG Ab. α‐Gal‐specific IgG_1–4_ Ab in individuals with and without meat allergy were assessed by ELISA.

**Results:**

In immunoblots, BGG was the most frequently recognized meat protein. Binding of IgE and IgG to BGG was confirmed by ELISA and completely abolished after pre‐incubation with α‐gal. Neither the depletion of autologous α‐gal‐specific IgG Ab nor the addition of α‐gal‐specific IgG Ab from nonallergic individuals changed the IgE recognition of BGG of meat‐allergic patients. Meat‐allergic patients showed significantly higher α‐gal‐specific IgG1 and IgG3 Ab than nonallergic individuals, whereas the latter showed significantly higher levels of α‐gal‐specific IgG4 Ab.

**Conclusion:**

Patients with delayed meat allergy display IgE and IgG Ab that selectively recognize the α‐gal epitope on BGG. Their enhanced α‐gal‐specific IgE levels are accompanied by high levels of α‐gal‐specific IgG1 devoid of IgE‐blocking activity. This subclass distribution is atypical for food allergies and distinct from natural α‐gal IgG responses in nonallergic individuals.

As for other food allergies, IgE responses to meat proteins have been detected in children experiencing hypersensitivity reactions upon consumption of beef 1, 2. Most allergic reactions have been reported to be directed against bovine serum albumin (BSA) 3; however, bovine gamma globulin (BGG) and myoglobin have also been identified as allergens 1, 2, 4, 5. In 2009, a different form of meat allergy was first reported that is mediated by IgE antibodies (Ab) specific for the carbohydrate gal‐α‐1,3‐gal‐β‐1,4‐GlcNAc (α‐gal) and characterized by symptoms ranging from generalized urticaria to anaphylaxis, typically starting 3–7 h after eating beef, pork or lamb 6, 7. The same patients tolerized chicken, turkey or fish because poultry and fish do not express α‐gal, a carbohydrate common in nonprimate mammals. Most reports on this new form of α‐gal‐mediated delayed‐type meat allergy included individuals living in south‐eastern regions of the United States 8, 9, 10. These data were complemented by reports on Australian and more lately also Swedish patients 11, 12. Anaphylaxis to pork kidney in France, Germany and Luxembourg has also been attributed to α‐gal‐specific IgE Ab 13, 14, 15. In addition to its involvement in delayed meat allergy, α‐gal has also been found to be a dominant IgE epitope on IgA from cats 16.

We obtained sera from 20 Austrian patients collected in allergy clinics over the past 10 years who had experienced severe, delayed hypersensitivity reactions upon consumption of red meat. We confirmed their IgE reactivity to α‐gal and employed them to identify α‐gal‐carrying proteins in beef. Immunoblots with meat extract revealed BGG as most prominent protein recognized by these patients. As BGG has been shown to be recognized by IgE Ab from children with meat allergy 1, we investigated whether BGG recognition by IgE from patients with delayed meat allergy involved amino acid residues or targeted the glycan structure only.

Children sensitized to egg and/or milk but tolerating these foods have been shown to display higher levels of allergen‐specific IgG4 and higher IgG4/IgE ratios than those with allergic reactions 17, 18, 19. Similarly, we have found allergen‐specific IgG4/IgE ratios to be higher in adult birch pollen‐allergic patients tolerant to foods like apple and hazelnut as compared to those suffering from birch pollen‐related food allergy 20. Together, these data have indicated that allergen‐specific IgG4 Ab play a role in food tolerance. Indeed, sera from IgG4‐positive food‐tolerant patients possessed IgG‐dependent IgE inhibitory activity 20. As all humans display high levels of α‐gal‐specific IgG Ab 21, we compared α‐gal‐specific IgG levels and subclasses in meat‐allergic and nonallergic individuals and assessed whether they affected IgE binding to α‐gal.

## Methods

### Study population

Sera from two patients with immediate and 20 patients with delayed allergic reactions to meat were included. Characteristics of the latter are described in Table 1. Total and allergen‐specific IgE levels were determined by ImmunoCAP (Thermo Fisher Scientific, Uppsala, Sweden). Nonallergic individuals (*n* = 20) did not report any allergic symptoms and showed no allergen‐specific IgE Ab (data not shown). Patients with birch pollen‐related apple allergy (*n* = 20) were previously described 22. Briefly, birch pollen‐related apple allergy was based on case history, positive skin prick tests, allergen‐specific IgE (>0.35 kU_A_/l, ImmunoCAP, Thermo Fisher Scientific) and oral provocation tests 22. None of the allergic patients underwent allergen‐specific immunotherapy. Approval was obtained from the ethics committee of the Medical University of Vienna (EK Nr.: 1162/2012).

**Table 1:** Clinical characterization of Austrian patients with delayed meat allergy

Patient no.	Age (y)	Sex	Total IgE (kU/l)	IgE [kU_A_/l] specific for				Clinical symptoms
				Beef	Pork	Chicken	α‐gal	
1	59	m	1034	40.3	26.0	<0.35	>100	ANA*, DYS, Itch, U
2	55	f	593	11.9	10.8	<0.35	n.t.	GI, U
3	86	f	745	14.4	11.0	<0.35	n.t.	U
4	70	m	502	36.5	32.3	<0.35	77.0	Itch on extremities, U
5	55	f	162	8.95	6.14	<0.35	61.3	AE, ANA, U
6	42	f	342	20.7	13.6	<0.35	37.4	U
7	68	m	146	3.93	3.48	<0.35	5.6	DYS, GI, Itch
8	33	f	337	3.81	1.89	<0.35	23.1	ANA, DYS, U
9	46	m	111	2.00	1.22	n.t.	2.3	ANA
10	30	f	535	7.59	0.72	0.78	n.t.	DYS
11	42	m	595	22.9	4.60	<0.35	n.t.	ANA, GI, Itch
12	81	f	115	9.71	11.9	<0.35	26.6	ANA, U
13	56	f	424	19.3	18.6	<0.35	>100	U
14	75	f	4030	0.94	0.62	<0.35	11.5	AE
15	41	f	880	0.46	0.84	0.38	n.t.	ANA, U
16	19	f	483	<0.35	2.84	<0.35	n.t.	AE, U
17	45	f	552	<0.35	2.65	<0.35	n.t.	GI, U
18	70	m	345	4.06	3.42	<0.35	99.9	U
19	32	f	37.3	1.05	0.98	<0.35	4.1	AE, GI, U
20	49	m	312	n.t.	n.t.	n.t.	>100	AE, ANA, U

AE, Angioedema; ANA, anaphylaxis; DYS, dyspnoea; f, female; GI, gastrointestinal symptoms; m, male; U, urticaria; y, years; n.t., not tested.

### Immunoblot experiments

Beef was purchased at a local butcher's store, shock‐frozen with liquid nitrogen, reduced to small pieces with a mortar and stirred in PBS containing protease inhibitors (Roche Diagnostics GmbH, Rotkreuz, Switzerland) overnight at 4°C. Thereafter, the extract was centrifuged at 10 000 g for 30 min and the supernatant was filtered through filter paper (Macherey‐Nagel, Düren, Germany), lyophilized and stored at −20°C. The protein concentration was determined by bicinchoninic acid assay (Bio‐Rad Laboratories, Richmond, CA, USA). The extract (20 μg) was separated by 12% SDS‐PAGE under nonreducing conditions and stained with Coomassie brilliant blue (Bio‐Rad Laboratories). Detection of glycosylation was performed with the Pro‐Q^®^ Emerald 300 Glycoprotein Gel and Blot Stain Kit (Thermo Fisher Scientific) according to the manufacturers’ protocol. For immunoblot experiments, the separated extract was transferred to a nitrocellulose membrane. After blocking, sera were incubated overnight at 4°C. Bound IgE was detected with ^125^I‐labelled anti‐human IgE antibody (Demeditec Diagnostics, Kiel‐Wellsee, Germany) and visualized by autoradiography. Buffer and sera of nonallergic donors served as negative controls.

### Antibody responses

Microtiter plates (Maxisorp, Nunc, Denmark) were coated with either Galα1‐3Galβ1‐4GlcNAc‐BSA (5 μg/ml; Dextra Laboratories, Reading, UK), BSA (5 μg/ml), BGG (400 μg/ml, both >99% pure and from Sigma‐Aldrich, Steinheim, Germany) or recombinant Mal d 1 (2 μg/ml; Biomay AG, Vienna, Austria). Optimal coating concentrations of each protein had been defined in preliminary experiments. Allergen‐coated plates were washed twice and saturated with 1% HSA in PBS/0.05% Tween‐20 for 6 h at room temperature. Subsequently, sera (diluted 1 : 5 in PBS; 0.05% Tween‐20; 0.5% HSA for IgE; 1 : 50 for IgG; and 1 : 20 for IgG_1–4_ detection) were incubated overnight at 4°C in duplicate. Buffer controls were done in sixfold replicates. After five washing steps, bound IgE and IgG Ab were determined with AP‐conjugated anti‐human IgE (BD Bioscience, Pharmingen, San Diego, CA, USA) and HRP‐conjugated goat anti‐human IgG (Jackson, West Grove, PA, USA), respectively. IgG_1–4_ Ab were analysed with anti‐human IgG1 (Sigma‐Aldrich), IgG2, IgG3 and IgG4 (all from BD Bioscience) and visualized with HRP‐conjugated anti‐mouse IgG (GE Healthcare, Vienna, Austria).

In inhibition experiments, sera were pre‐incubated with indicated concentrations of BGG, α‐gal‐BSA or BSA for 6 h at room temperature. Moreover, plate‐bound BGG was incubated with sera overnight at 4°C prior to the addition of sera from meat‐allergic patients. Thereafter, reactivity of IgE and IgG to plate‐bound BGG was determined as described above.

For IgG depletion, sera were incubated with protein G Sepharose (Bio Vision, Milpitas, CA, USA) for 4 h at room temperature. Sephadex G‐50 (Sigma‐Aldrich) served as negative control. After centrifugation, protein G Sepharose and Sephadex G‐50 were added to the supernatant and again incubated. This procedure was repeated four times. To destroy the binding of IgE Ab, sera were incubated for 30 min at 56°C.

### Statistical analysis

Statistical differences were determined using SPSS 20.0 (Chicago, IL, USA). Differences were considered statistically significant for *P* < 0.05. Data from duplicate and triplicate experiments are shown as means with standard deviation (SD).

## Results

### Clinical characterization of meat‐allergic patients

Data on age (median 55, range 19–86 years), sex (13 female, six male), total and specific IgE levels for beef, pork and chicken of 20 Austrian patients are summarized in Table 1. These individuals had experienced meat‐induced symptoms occurring 3–7 h after ingestion that comprised angioedema, diarrhoea, drop of blood pressure, dyspnoea, exanthema, itchiness, nausea, swelling, tachycardia, unconsciousness, urticaria or vomiting (Table 1). Patients displayed IgE specific for either beef or pork extract. Two patients displayed low IgE levels specific for chicken extract. IgE reactivity to α‐gal was assessed in 13 patients by ImmunoCAP (Table 1) and in 19 patients by ELISA (Table 2).

**Table 2:** IgE reactivity of patients with delayed meat allergy to BGG, α‐gal and BSA

Patient no.	IgE ELISA (O.D.)			IgE [kU_A_/l]	Patient no.	IgE ELISA (O.D.)			IgE [kU_A_/l]
	BGG	α‐gal‐BSA	BSA	α‐gal		BGG	α‐gal‐BSA	BSA	α‐gal
1	1.092*	>2.5*	0.053†	>100	12	0.280	1.174	0.050	26.6
2	0.214	1.604	0.059	n.t.	13	0.705	>2.5	0.068	>100
3	0.535	1.402	0.059	n.t.	14	0.134	0.664	0.061	11.5
4	1.616	1.018	0.081	77.0	15	0.372	>2.5	0.127	n.t.
5	0.275	0.584	0.044	61.3	16	0.394	1.535	0.096	n.t.
6	0.328	0.549	0.044	37.4	17	0.201	0.306	0.138	n.t.
7	0.189	0.320	0.044	5.6	18	1.113	>2.5	0.191	99.9
8	0.311	0.178	0.050	23.1	19	0.166	0.089	0.046	4.1
9	0.258	0.525	0.105	2.3	N1	0.084	0.065	0.050	<0.35
10	0.142	0.557	0.104	n.t.	N2	0.121	0.080	0.070	<0.35
11	n.t.	1.582	0.150	n.t.	B	0.066‡	0.050‡	0.049‡	n.t.

O.D., optical density; *mean O.D. of duplicates, †mean O.D. of duplicates plus 5* standard deviation; ‡mean O.D. of 6 values plus 5* standard deviation; N1 and N2, nonallergic controls; B, buffer control; n.t., not tested.

### Meat‐allergic patients show IgE reactivity to bovine gamma globulin

**Figure 1:**
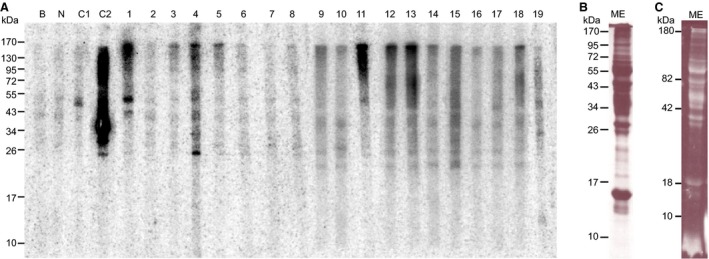
IgE reactivity patterns of 19 patients with delayed meat allergy. (A) Meat extract (ME) was separated under nonreducing conditions by SDS‐PAGE, transferred to nitrocellulose and incubated with patients′ sera. Human IgE was detected by autoradiography; (B) proteins visualized by Coomassie staining; (C) glycosylated proteins; B, buffer control; N, nonallergic control; C1, C2, patients with immediate reactions to red meat.

To identify bovine proteins recognized by IgE Ab of patients with delayed meat allergy, we analysed sera from 19 patients in immunoblots (Fig. 1A). Nonallergic individuals and the buffer control showed no IgE reactivity. Sera from two patients with immediate allergic reactions to beef (C1 and C2) served as positive controls. C1 selectively detected the major allergen BSA and C2 confirmed the presence of various additional allergenic proteins in our meat extract, respectively. However, except for patient nos 1 and 4, none of the patients with delayed meat‐induced reactions showed well‐defined intense bands to any of the proteins detected by protein staining (Fig. 1B). Instead, all individuals showed several weak and blurred bands and patients nos 1, 3–6, 8–14 and 18 recognized a protein of high molecular weight (>150 kDa), presumably BGG (160 kDa, Fig. 1A). We could confirm IgE reactivity to pure BGG in 18 patients by ELISA (Table 2). The O.D. values to α‐gal‐BSA and BSA are shown for comparison.

### BGG‐reactive IgE and IgG Ab of meat‐allergic patients react selectively with α‐gal

**Figure 2:**
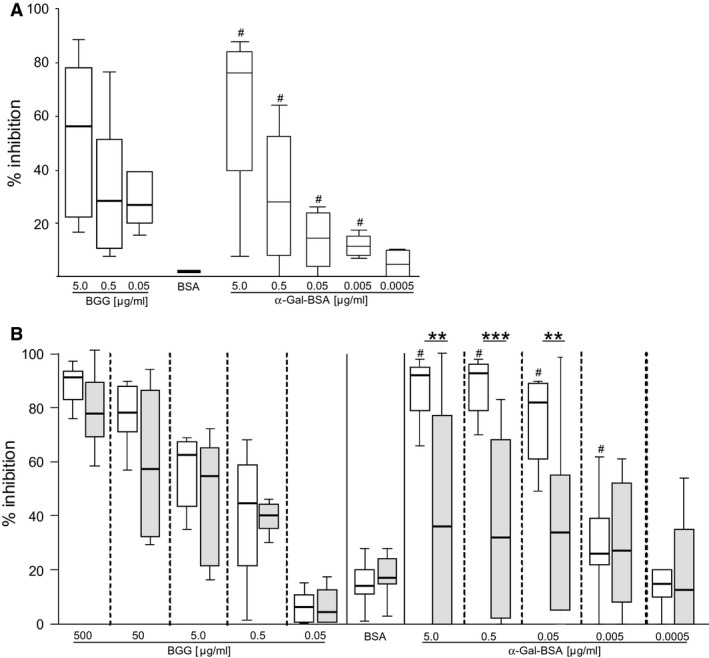
Inhibition of IgE and IgG binding to BGG by α‐gal. (A) Sera from four and (B) eight meat‐allergic (white box plots) and eight nonallergic (grey box plots) individuals were pre‐incubated with indicated amounts of BGG, BSA or α‐gal‐BSA. The percentage of inhibition of (A) IgE and (B) IgG binding to BGG is shown. ^#^ *P* < 0.05 compared to BSA; **P < 0.01 and ***P < 0.001 (Wilcoxon signed rank test).

Next, we sought to evaluate whether BGG‐specific Ab of patients with delayed meat allergy were directed against α‐gal or an epitope consisting of both the carbohydrate and amino acid residues. For this purpose, sera from four meat‐allergic patients were incubated with titrated concentrations of BGG, BSA or α‐gal‐BSA and thereafter tested for IgE reactivity to BGG (Fig. 2A). In addition, we analysed the inhibition of IgG binding to BGG in sera from eight patients with delayed meat allergy and eight non‐meat‐allergic individuals for comparison (Fig. 2B). IgE and IgG binding to BGG in meat‐allergic patients was strongly and dose dependently inhibited by α‐gal‐BSA. Concentrations of 5, 0.5, 0.05 and 0.005 μg/ml α‐gal‐BSA reduced IgE and IgG binding to BGG significantly stronger than 5 μg/ml of BSA. However, the inhibition pattern in non‐meat‐allergic individuals looked notably different. In contrast to self‐inhibition by BGG, the reduction in IgG binding to BGG by α‐gal‐BSA varied individually, which was overall neither dose dependent nor significantly different from BSA and significantly lower than in patients with delayed meat allergy (Fig. 2B).

### α‐Gal‐specific IgG Ab do not affect IgE binding to BGG in meat‐allergic patients

**Figure 3:**
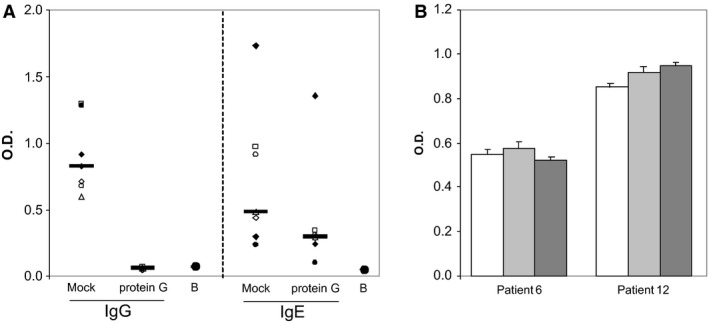
α‐Gal‐specific IgG does not block IgE binding to BGG. (A) Sera from seven meat‐allergic patients were either mock‐treated or incubated with protein G. Thereafter, binding of IgG and IgE to BGG was assessed. Black lines indicate median values. B, buffer control; O.D. optical density; (B) plate‐bound BGG was incubated with buffer (white bars), sera from patient 6 and 12 incubated for 30 min at 56°C (light grey bars) or pooled sera from two nonallergic patients containing high levels of α‐gal‐specific IgG Ab (dark grey bars). Thereafter, untreated sera from patient 6 and 12 were added and BGG‐specific IgE was assessed.

To assess whether the high levels of α‐gal‐specific IgG Ab affected IgE binding to the carbohydrate, sera from seven meat‐allergic patients were either mock‐treated or depleted from IgG by repeated incubation with protein G. Thereafter, IgE binding to BGG was assessed by ELISA. Figure 3A shows that treatment with protein G completely reduced BGG‐specific IgG Ab in all sera. However, the absence of IgG Ab did not result in enhanced IgE reactivity to BGG. To address whether BGG‐specific IgG Ab from nonallergic individuals might show blocking activity, we took a different experimental approach. Plate‐bound BGG was saturated with pooled sera from two nonallergic individuals with high α‐gal‐specific IgG levels or sera from two allergic patients after incubation for 4 h at 56°C. This treatment destroyed IgE but not IgG Ab to α‐gal (data not shown), thereby preventing the blocking of IgE by IgE Ab. Thereafter, untreated sera from the same meat‐allergic individuals were added and IgE binding to BGG was assessed. Figure 3B shows that neither the sera from nonallergic individuals nor autologous heat‐treated sera reduced IgE binding to BGG.

### IgG1 is the predominant α‐gal‐specific IgG subclass in meat‐allergic patients

**Figure 4:**
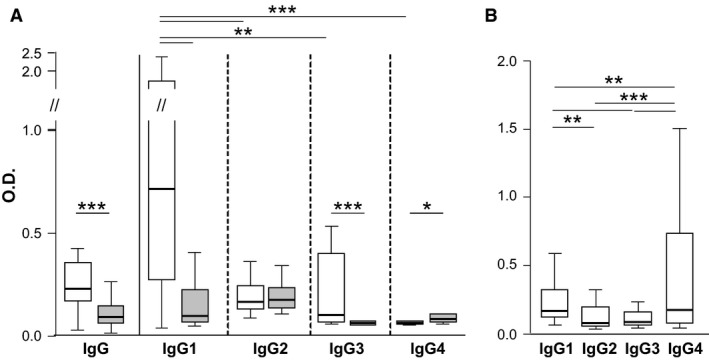
α‐Gal‐specific IgG responses in food‐allergic and nonallergic individuals. (A) α‐Gal‐specific IgG_1–4_ in 18 meat‐allergic (white) and 20 nonallergic (grey) individuals, (B) Mal d 1‐specific IgG_1–4_ in 20 patients with birch pollen‐related apple allergy; O.D. optical density, *P < 0.05, **P > 0.01, ***P > 0.001 (Wilcoxon signed rank test).

We next compared the subclass distribution of α‐gal‐specific IgG Ab in meat‐allergic and nonallergic individuals. Patients (*n* = 18) with delayed meat allergy displayed significantly higher levels of α‐gal‐specific total IgG (*P* < 0.001), IgG1 (*P* = 0.002) and IgG3 (*P* = 0.003) than nonallergic individuals (*n* = 20, Fig. 4A). In contrast, nonallergic individuals showed significantly higher levels of α‐gal‐specific IgG4 than meat‐allergic patients (*P =* 0.03). α‐Gal‐specific IgG2 levels did not differ in both groups.

Among meat‐allergic individuals, α‐gal‐specific IgG1 was significantly higher than IgG2‐4 values (*P* < 0.001, *P* = 0.002 and *P* < 0.001, respectively). Moreover, α‐gal‐specific IgG2 and IgG3 values were significantly higher than α‐gal‐specific IgG4 values (*P* < 0.001 and *P* = 0.002, respectively). For comparison, we tested sera from 20 patients with birch pollen‐related apple allergy 22. These patients displayed significantly higher IgG4 than IgG1‐3 values specific for the major apple allergen Mal d 1 (*P* = 0.012, *P* < 0.001 and *P* < 0.001, respectively). Furthermore, Mal d 1‐specific IgG1 was significantly higher than IgG2 and IgG3 values (*P* = 0.014 and *P* < 0.001, Fig. 4B).

## Discussion

α‐Gal‐mediated delayed hypersensitivity to red meat has several unusual characteristics. Although IgE‐mediated, the symptoms occur atypically late for immediate hypersensitivity, namely 3–7 h after ingestion of red meat 23. The major allergen is not a protein but a mammalian oligosaccharide and removal of α‐gal dramatically reduces IgE binding to beef proteins 24. Several studies provided evidence that sensitization to α‐gal results from tick bites 11, 12, 25, 26. Consequently, delayed meat allergy is not a consequence of oral but cutaneous sensitization to the α‐gal epitope expressed on proteins present in the gastrointestinal tract of these arthropods 27. Thereafter, individuals may develop allergic reactions due to IgE reactivity with α‐gal on mammalian proteins, for example murine and feline Ig 16, 24, 28. This pathophysiology is reminiscent of other, so‐called secondary food allergies, for example birch pollen‐related food allergy. Here, sensitization occurs to Bet v 1 *via* the respiratory tract and allergic reactions to foods are caused by IgE reactivity to similar conformational epitopes on structurally related food proteins, for example Mal d 1 in apple 29. We present 20 Austrian patients with delayed meat allergy and IgE reactivity to α‐gal on bovine IgG. After 39 Swedish patients 12, this is the second largest European cohort described up to now. Similar to the Swedish group (median age of 52 years, range 18–74 years) and recently described patients from the United States (median age of 42 years, range 19–55 years) 23, the median age of our patients was 55 years (range 19–86 years). Hence, α‐gal‐mediated delayed‐type meat allergy manifests later in life which is another characteristic of secondary food allergy.

We used our study cohort to identify α‐gal‐carrying proteins in beef because to the best of our knowledge, only five sera from patients with α‐gal‐mediated delayed meat allergy have been employed to address this question so far 30. Those patients showed multiple but overall faint bands in immunoblots. The IgE‐binding patterns of 17 of our 19 patients looked similar; however, 68% of them apparently recognized BGG (Fig. 1). IgE recognition of BGG by all patients was confirmed by ELISA (Table 2). To address whether IgE epitopes on BGG involved amino acid residues or the glycan only, we performed inhibition experiments with α‐gal to avoid possible defolding of BGG by deglycosylation. IgE recognition of BGG was completely abolished by α‐gal conjugated to BSA (Fig. 2) confirming that patients with delayed meat allergy selectively recognize α‐gal independently from the carrier protein 24.

In addition, the inhibition experiments indicated that the epitope specificity of α‐gal‐specific IgG Ab differed in meat‐allergic and nonallergic individuals. In meat‐allergic patients, the IgG reactivity to BGG was concordantly blocked by α‐gal, whereas the inhibition pattern in nonallergic individuals showed high individual variability and was neither dose dependent nor significantly different from the negative control. Thus, BGG recognition by nonallergic individuals involved amino acid residues in addition to α‐gal, in contrast to the exclusive recognition of the glycan by meat‐allergic patients. We detected significantly higher levels of BGG‐specific (*P* = 0.004, data not shown) and α‐gal‐specific IgG (Fig. 4A) Ab in meat‐allergic than in nonallergic individuals and depleted these Ab from their sera. However, the absence of α‐gal‐specific IgG did not enhance α‐gal‐specific IgE binding. To assess whether BGG‐specific IgG Ab from nonallergic individuals with different epitope specificity exerted potential blocking capacity, we saturated plate‐bound BGG with their sera. In parallel, sera from meat‐allergic patients lacking functional IgE Ab after heat treatment were used. Neither autologous nor heterologous α‐gal‐specific IgG Ab from nonallergic individuals were able to prevent IgE binding to the carbohydrate.

Finally, we were curious about the subclass distribution of α‐gal‐specific IgG Ab in patients with delayed meat allergy because α‐gal‐specific IgG1 and IgG2 and not IgG4 Ab were previously reported to accompany IgE Ab in α‐gal‐sensitized individuals 31. In our atopic (median value of total IgE levels: 483 kU/l, range 37.3 – 4030 kU/l) cohort with delayed meat allergy IgG1 represented the predominant α‐gal‐specific subclass. This subclass distribution differs from IgG responses to BSA reported in meat‐allergic children 32 and from non‐meat‐allergic individuals (Fig. 4A). Moreover, this subclass distribution is distinct from other secondary food allergies as IgG4 Ab dominated the Mal d 1‐specific IgG response in patients with birch pollen‐related apple allergy (Fig. 4B).

In summary, patients with α‐gal‐mediated delayed meat allergy display α‐gal‐specific IgG responses atypical for food allergies and distinct from natural α‐gal responses in non‐meat‐allergic individuals. Although specific for the same epitope, the high levels of α‐gal‐specific IgG1 in meat‐allergic patients did not interfere with IgE binding to the carbohydrate. This observation may be referred to the low avidity of anticarbohydrate Ab or to the lower affinity of IgG compared to IgE Ab 33. Further studies are currently being performed to address these issues.

## Author contributions

D.K. and B.B. designed the experiments; D.K., B.N. and C.K.; C.E., W.E., S.W. and S.V. provided patients′ samples; A.M. and H.J.A. provided experimental protocols; and D.K. and B.B. wrote the manuscript.

## Conflicts of interest

The authors declare that they have no conflicts of interest.
